# Self-perceived Fracture Risk in the Global Longitudinal Study of Osteoporosis in Women: Its Correlates and Relationship with Bone Microarchitecture

**DOI:** 10.1007/s00223-020-00680-9

**Published:** 2020-03-05

**Authors:** A. E. Litwic, L. D. Westbury, S. Carter, K. A. Ward, C. Cooper, E. M. Dennison

**Affiliations:** 1 MRC Lifecourse Epidemiology Unit, University of Southampton, Southampton, UK; 2 Department of Nephrology, Transplantology and Internal Medicine, Medical University of Gdańsk, Gdańsk, Poland; 3 MRC Nutrition and Bone Health Research Group, Cambridge, UK; 4 NIHR Southampton Biomedical Research Centre, University of Southampton and University Hospital Southampton NHS Foundation Trust, Southampton, UK; 5 NIHR Oxford Biomedical Research Centre, University of Oxford, Oxford, UK; 6 Victoria University of Wellington, Wellington, New Zealand

**Keywords:** Self-perceived fracture risk, Determinants, HRpQCT, DXA, Osteoporosis, Epidemiology, Fracture risk assessment

## Abstract

The purpose of this study is to examine correlates of self-perceived fracture risk (SPR) and relationships between SPR and subsequent bone density and microarchitecture in the UK arm of the Global Longitudinal Study of Osteoporosis in Women. 3912 women completed baseline questionnaires detailing medical history and SPR; 492 underwent HRpQCT scans of the radius and tibia and DXA scans of total body, hip, femoral neck and lumbar spine a median of 7.5 years later. Correlates of SPR were examined and a cluster analysis of potential predictors of SPR performed. SPR in relation to HRpQCT and aBMD parameters was examined using linear regression with and without adjustment for anthropometric, demographic and lifestyle covariates. Mean (SD) baseline age was 69.0 (9.0) years; 56.6% reported a similar SPR; 28.6% lower SPR; 14.9% higher SPR compared to women of similar age. In mutually-adjusted analysis, higher SPR was associated (*p* < 0.05) with: lower physical activity and educational attainment; use of anti–osteoporosis medications (AOM) and calcium supplements; greater number of falls in the previous year; history of fracture since aged 45; family history of hip fracture; and increased comorbidity. Higher SPR, history of fracture, and use of AOM, calcium and vitamin D clustered together. Even after adjustments that included AOM use, higher SPR was associated with: lower radial trabecular volumetric density and number, and higher trabecular separation; lower tibial cortical area and trabecular volumetric density; and lower aBMD at the femoral neck. Despite greater AOM use, women with higher baseline SPR had poorer subsequent bone health.

**Electronic supplementary material:**

The online version of this article (10.1007/s00223-020-00680-9) contains supplementary material, which is available to authorized users.

## Introduction

Osteoporosis, a disease characterised by low bone mass and structural deterioration, is classified as a public health problem due to its association with an increased risk for fragility fractures and, consequently has a high impact on quality of life and high rates of morbidity [1]. Worldwide, there are nearly nine million osteoporotic fractures each year, with reports suggesting that one in two women and one in five men will experience a fracture in their remaining lifetime from the age of 50 years [1, 2]. With ageing of the population, the economic cost of osteoporosis and fractures is projected to increase in the EU from €37.4 billion in 2010 to €46.8 billion by 2025 and, in the US, from $17 billion in 2005 to $25.3 billion by 2025 [3, 4].

Patient and healthcare provider awareness of individual fracture risk is essential for accurate planning and successful implementation of prevention strategies. A number of web-based tools have been developed to improve the identification of individuals at high fracture risk. Clinical risk factors such as age, weight and skeletal properties are included in fracture prediction algorithms, with the most commonly used globally being FRAX. Recently, it has been reported in the Global Longitudinal Study of Osteoporosis in Women (GLOW) that self-perceived fracture risk (SPR) may also capture aspects of fracture risk not measured using current risk prediction tools, and has been associated with fracture risk independently of FRAX [5].

Self-perception of risk of a condition is a difficult concept, as it requires an individual to compare their own health status to others. There is evidence that self-perception of risk of osteoporosis and osteoporotic fractures is underestimated in postmenopausal women worldwide [6], and that self-perceived risks of osteoporosis and fracture affect certain behaviours such as seeking medical advice, anti-osteoporosis medication use and BMD screening, which might lead to greater healthcare engagement, treatment and altered bone health [5, 7]. Furthermore, findings from the GLOW cohort suggest that increased self-perceived fracture risk is strongly associated with incident fracture rate [8]. However, very little is known about what determines self-perceived fracture risk.

To address this, we have used data from the UK arm of the GLOW to: identify correlates of SPR; examine how these correlates interrelate by performing a cluster analysis; and relate SPR to subsequent bone density and microarchitecture.

## Methods

### Study Participants

GLOW is a prospective, observational cohort study conducted through general physician practices in 10 countries. Study design and recruitment have been described in detail previously [9]. In brief, practices, representative of each region, were recruited through primary care networks and provided the names of women aged 55 years and older who had been seen by their physician in the past 24 months. The primary aim of GLOW was to characterise the descriptive epidemiology and health impact of osteoporosis-related fractures among women who were 55 years of age and older worldwide. Globally, GLOW enrolled over 60,000 women through over 700 physicians in 10 countries, and conducted annual follow-up for up to 5 years. In Southampton only, a subgroup of participants with baseline data and at least one follow-up questionnaire were invited, after completion of 5 years of follow-up, for a follow-up study which included dual-energy X-ray absorptiometry (DXA) and high resolution peripheral quantitative computed tomography (HRpQCT) scans. Participants were scanned between April 2014 and December 2017. Patients, who were institutionalized or were not able to complete the study survey by themselves due to cognitive impairment, language barriers, institutionalization, or were too ill to complete the survey or attend for the scans were excluded.

### Baseline Questionnaires

To ascertain self-perceived fracture risk (SPR), participants were asked to rate their risk of fracturing/breaking a bone, compared to other women of the same age, out of the following responses: ‘much lower’; ‘a little lower’; ‘about the same’; ‘a little higher’; and ‘much higher’. Fracture history since age 45 years was ascertained at the following locations: clavicle, upper arm, wrist, spine, rib, hip, pelvis, ankle, upper leg and lower leg. Family history of hip fracture was obtained by asking participants whether their mother or father had ever broken or fractured their hip. Information on the number of falls during the previous 12 months was also collected.

Further information ascertained from questionnaires included: age; self-reported height and weight; smoking status; alcohol consumption; physical activity; educational attainment; current use of anti-osteoporotic medication (AOM), calcium supplements and Vitamin D supplements (or multivitamin with Vitamin D); current/previous use of oestrogen or hormone replacement therapy (HRT); and years since menopause. Participants were considered to be taking AOM if they reported current use of alendronate, calcitonin, etidronate, ibandronate, pamidronate, raloxifene, risedronate, strontium ranelate, teriparatide, tibolone or zoledronic acid. Participants were asked whether a doctor or health provider had ever told them that they had the following conditions: hypertension; heart disease; high cholesterol; asthma; chronic bronchitis/emphysema; osteoporosis; osteoarthritis/degenerative joint disease; rheumatoid arthritis; stroke; ulcerative colitis/Crohn’s disease; celiac disease; Parkinson’s disease; multiple sclerosis; cancer; and type 1 diabetes.

### Anthropometry and DXA

In a subgroup of participants that underwent DXA at a median (lower quartile, upper quartile) of 7.5 (7.1, 8.9) years after the baseline questionnaire, height was measured to the nearest 0.1 cm using a Marsden stadiometer on the day of scanning; weight was measured to the nearest 0.1 kg using a Marsden MPPS-250 (Marsden Weighing Machine Group Limited, Rotherham, UK) digital floor scale. Areal bone mineral density (aBMD, g/cm^2^) of the total body, hip, femoral neck and lumbar spine was measured using a DXA Hologic Horizon W (software version Apex 5.5.3.1 [Vertec Scientific, Reading, UK]).

### Assessment of Bone by HRpQCT

This subgroup of participants also underwent a HRpQCT scan of the non-dominant distal radius and tibia using XtremeCT I (Scanco Medical, Basserdorf, Switzerland) on the same day as the DXA scan; if there was a history of fracture on the non-dominant limb, the non-fractured limb was measured. A stack of 104 parallel HRpQCT slices were acquired with an isotropic voxel size of 82 µm. Methods used to process the HRpQCT data have been described previously [10]. For this analysis, the standard evaluation and cortical porosity scripts were run to obtain estimates of the following parameters at the radius and tibia: total area and trabecular area, volumetric density, number, thickness and separation; cortical area, thickness, volumetric density and pores diameter; and cortical porosity [11].

### Derived Variables

Self-reported body mass index (BMI) at baseline was calculated from the self-reported measures of height and weight. Self-reported height and weight were correlated (*r* = 0.32, *p* < 0.001); a sex-specific standardised residual of weight-adjusted-for-height at baseline was derived as a marker of adiposity for inclusion in regression models. Variables for BMI and weight-for-height residual were also calculated at follow-up from measured height and weight among the subgroup that underwent DXA and HRpQCT. The total number of comorbidities at baseline, excluding osteoporosis, was used as a marker for overall morbidity. FRAX scores for 10-year probability of major osteoporotic fracture (MOF) and hip fracture were calculated for women from their baseline survey responses, without inclusion of bone mineral density measurements.

### Statistical Analysis: Cross-Sectional Correlates of SPR at Baseline

Participant characteristics of the 3912 women with data on SPR at baseline were described using summary statistics (Table 1). Ordinal logistic regression was used to examine univariate associations between participant characteristics and SPR. Characteristics significantly associated (*p* < 0.05) with SPR were then included in a mutually-adjusted model; FRAX scores were not included in mutually-adjusted analyses as the inclusion of these variables and participant characteristics which are components of FRAX may result in multicollinearity. Sensitivity analyses were performed among the following groups; have osteoporosis; current use of AOM; have osteoporosis or current use of AOM.

**Table 1:** Baseline participant characteristics of the analysis sample (*n* = 3912)

Participant characteristic	*N *(%)	Missing values
Age (years)*	69.0 (9.0)	0
Self-reported height (cm)*	161.7 (6.8)	193
Self-reported weight (kg)*	68.3 (12.8)	215
BMI (kg/m^2^)*	26.1 (4.7)	354
Current smoker	273 (7.1%)	48
Self-perceived fracture risk		
Much lower	472 (12.1%)	0
A little lower	646 (16.5%)	
About the same	2213 (56.6%)	
A little higher	442 (11.3%)	
Much higher	139 (3.6%)	
Alcohol consumption		
None	1242 (32.0%)	34
1–6	1598 (41.2%)	
7–13	779 (20.1%)	
14–20	222 (5.7%)	
> 20	37 (1.0%)	
Physically active compared to others		
Not at all	135 (3.5%)	56
A little	694 (18.0%)	
Somewhat	1893 (49.1%)	
Very	1134 (29.4%)	
Educational attainment		
Below GCSE	1540 (39.4%)	0
GCSE	1185 (30.3%)	
A-level	522 (13.3%)	
Degree	665 (17.0%)	
Current use of anti-osteoporotic medication	348 (9.4%)	222
Ever used oestrogen/hormone replacement therapy	1328 (34.6%)	71
Currently taking calcium	736 (19.3%)	97
Currently taking Vit D/multivitamin with Vit D	695 (18.2%)	103
Years since menopause		
Less than 10 years	677 (17.8%)	98
10–19 years	1195 (31.3%)	
20–29 years	1050 (27.5%)	
30 or more years	892 (23.4%)	
Falls in previous 12 months		
None	2394 (61.9%)	44
Once	902 (23.3%)	
2 times or more	572 (14.8%)	
Fracture since 45 years	763 (20.5%)	182
Family history of hip fracture	490 (14.3%)	489
FRAX 10-year probability (MOF)^†^	10.9 (7.3, 17.6)	1359
FRAX 10-year probability (hip fracture)^†^	2.2 (1.1, 5.9)	1359
Osteoporosis	413 (10.9%)	138
Number of comorbidities		
0	840 (24.9%)	543
1	1002 (29.7%)	
2	826 (24.5%)	
3	445 (13.2%)	
4 or more	256 (7.6%)	

*Mean (SD), ^†^Median (lower quartile, upper quartile)

*MOF* major osteoporotic fracture

### Statistical Analysis: Cluster Analysis of Potential Predictors of SPR

**Fig. 1:**
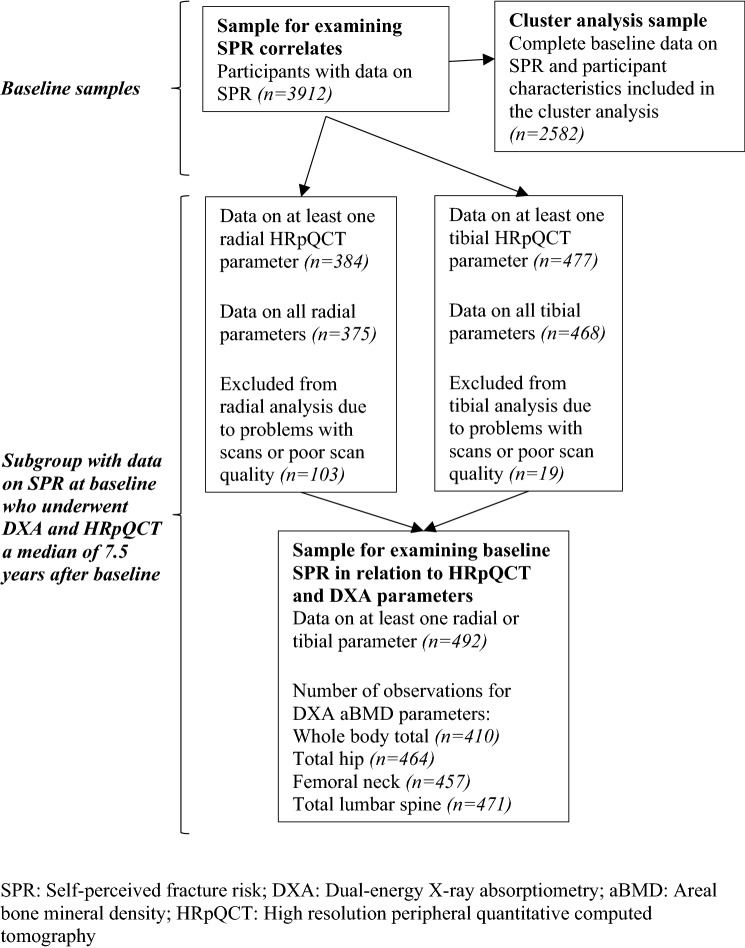
Flow diagram for the analytical samples of participants

A cluster analysis of the participant characteristics in Table 1 (excluding SPR, osteoporosis and FRAX probability variables and only using self-reported height and weight-for-height residual as measures of anthropometry) was performed among the 2582 participants with complete data on these characteristics; a flow diagram for the various samples of participants used for analysis is presented in Fig. 1. This used the TwoStep Cluster Analysis procedure in SPSS (version 25) which is suitable for a mixture of categorical and continuous variables [12]. This procedure involves grouping observations into clusters based on the distance measure and then applying a hierarchical clustering algorithm to these clusters; the cluster solution with the lowest Bayesian information criterion (BIC) is selected as optimal. The change in log-likelihood from merging two clusters as opposed to keeping them separate was used as the distance measure. Goodness-of-fit of the cluster solution was determined using the silhouette coefficient, a measure of how similar participants are within clusters compared to how similar they are between clusters, which ranges from − 1 to 1 (< 0.2: poor; 0.2–0.5: fair; > 0.5: good). Participant characteristics were then compared between the clusters using descriptive statistics.

### Statistical Analysis: SPR in Relation to DXA aBMD and HRpQCT Parameters

The sample for this subgroup analysis comprised 492 individuals with data on SPR and at least one of the HRpQCT parameters outlined above. Of these 492 participants, 384 and 477 had data on at least one radial and tibial HRpQCT parameter, respectively; the number of participants with available data for the DXA aBMD parameters ranged from 410 to 471, depending on the parameter (Fig. 1). Participant characteristics of this whole subgroup were described using summary statistics. Linear regression was used to examine SPR in relation to the HRpQCT parameters of the tibia and radius and the aBMD parameters. Unadjusted and adjusted associations, accounting for age at time of scan, follow-up time, measured height at follow-up, weight-for-height residual from measured values at follow-up, physical activity, smoking status, alcohol consumption, education, time since last menstrual cycle, use of AOM, calcium and vitamin D supplements, and oestrogen/HRT, were examined. SPR was treated as an ordinal variable with five levels. Apart from the cluster analysis, all analyses were conducted using Stata, version 15.0

## Results

### Participant Characteristics

Baseline participant characteristics of the baseline analysis sample (*n* = 3912) are presented in Table 1. Mean (SD) age was 69.0 (9.0) years. Overall, 2213 (56.6%) reported a similar SPR compared to other women of the same age; 1118 (28.6%) reported a lower risk and 581 (14.9%) reported a higher risk. Median (lower quartile, upper quartile) FRAX probabilities for 10-year MOF and hip fracture are presented in Table 1. MOF FRAX probabilities for women with lower, similar and higher SPR were 10.4 (7.1, 16.0), 10.7 (7.1, 17.2) and 15.6 (9.1, 22.8), respectively; corresponding FRAX probabilities for hip fracture were 2.1 (1.1, 5.4), 2.1 (1.0, 5.5) and 3.7 (1.6, 8.8) (data not shown).

Participant characteristics for the subgroup analysis sample (*n* = 492) who underwent bone assessments are presented in Table 2. Mean (SD) age at scan was 70.9 (5.4) years, resulting in a median (lower quartile, upper quartile) follow-up time of 7.5 (7.1, 8.9) years. Overall, 283 (57.5%) reported a similar SPR compared to other women of the same age; 140 (28.5%) reported a lower risk and 69 (14.0%) reported a higher risk.

**Table 2:** Baseline characteristics of subgroup who participated in bone phenotyping study (*n* = 492)

Participant characteristic	*N* (%)	Non-missing values
Age of SPR ascertainment*	63.0 (5.4)	492
Age at scan (years)*	70.9 (5.4)	489
Height (cm)*	160.3 (6.2)	482
Weight (kg)*	68.7 (12.7)	482
BMI (kg/m^2^)*	26.8 (5.0)	482
Whole body total aBMD (g/cm^2^)*	1.01 (0.10)	410
Total hip aBMD (g/cm^2^)*	0.84 (0.11)	464
Femoral neck aBMD (g/cm^2^)*	0.69 (0.10)	457
Total lumbar spine aBMD (g/cm^2^)*	0.92 (0.15)	471
Any fracture since 45 years	69 (14.4%)	478
Family history of hip fracture	63 (14.3%)	442
SPR compared to others		
Much lower	42 (8.5%)	492
A little lower	98 (19.9%)	
About the same	283 (57.5%)	
A little higher	58 (11.8%)	
Much higher	11 (2.2%)	
How active compared to others		
Not at all	7 (1.4%)	487
A little	69 (14.2%)	
Somewhat	241 (49.5%)	
Very	170 (34.9%)	
Current smoker	28 (5.7%)	487
Alcoholic drinks per week		
None	101 (20.6%)	490
1–6	208 (42.4%)	
7–13	131 (26.7%)	
14–20	39 (8.0%)	
> 20	11 (2.2%)	
Education		
Below GCSE	120 (24.4%)	492
CSE O level/GCSE	170 (34.6%)	
A-level	61 (12.4%)	
Degree	141 (28.7%)	
Use of anti-osteoporotic medication	31 (6.5%)	478
Currently taking calcium	101 (20.9%)	484
Currently taking Vit D/multivitamin with Vit D	112 (23.2%)	482
Ever used oestrogen/hormone replacement therapy	238 (48.6%)	490
Years since last menstrual cycle		
< 10	153 (31.9%)	479
10–19	212 (44.3%)	
20–29	89 (18.6%)	
> 29	25 (5.2%)	

^*^Mean (SD)

*SPR* self-perceived fracture risk, *DXA* dual-energy X-ray absorptiometry, *aBMD* areal bone mineral density

### Associations Between Baseline Participant Characteristics and SPR

Cross-sectional associations between baseline participant characteristics and SPR are presented in Table 3. In univariate analyses, the following were associated (*p* < 0.05) with higher SPR: shorter self-reported height; lower alcohol consumption, physical activity and educational attainment; current use of AOM and calcium supplements; longer time since menopause; greater number of falls in the previous 12 months; history of fracture since aged 45 years; family history of hip fracture; higher FRAX scores for MOF and hip fracture; and increased comorbidity. Apart from associations regarding self-reported height and alcohol consumption, all were significant (*p* < 0.05) in mutually-adjusted analysis (FRAX variables were not included in the mutually-adjusted model); however, the direction was reversed for time since menopause such that greater time was associated with reduced SPR.

**Table 3:** Odds ratios for having a higher category of self-perceived fracture risk for the presence versus absence of each characteristic

Characteristic	Univariate		Mutually-adjusted	
	Odds ratio (95% CI)	*p*-value	Odds ratio (95% CI)	*p*-value
Age*	1.02 (0.96, 1.08)	0.584		
Self-reported height*	**0.92 **(**0.86**, **0.97**)	**0.006**	0.95 (0.87, 1.02)	0.152
Weight-for-height residual*	1.02 (0.96, 1.09)	0.527		
Current smoker	1.11 (0.88, 1.41)	0.388		
Alcohol consumption**	**0.94 **(**0.88**, **1.00**)	**0.049**	1.04 (0.95, 1.13)	0.399
Physically active compared to others of similar age**	**0.52 **(**0.48**, **0.57**)	**< 0.001**	**0.52 **(**0.47**, **0.58**)	**< 0.001**
Educational attainment**	**0.90 **(**0.85**, **0.95**)	**< 0.001**	**0.87 **(**0.81**, **0.93**)	**< 0.001**
Current use of anti-osteoporotic medication	**8.99 **(**7.15**, **11.29**)	**< 0.001**	**6.10 **(**4.48**, **8.32**)	**< 0.001**
Ever used oestrogen/hormone replacement therapy	1.06 (0.94, 1.21)	0.345		
Currently taking calcium supplements	**3.04 **(**2.58**, **3.59**)	**< 0.001**	**1.64 **(**1.32**, **2.03**)	**< 0.001**
Currently taking Vit D/multivitamin with Vit D	1.09 (0.93, 1.28)	0.300		
Years since menopause**	**1.06 **(**1.00**, **1.13**)	**0.040**	**0.82 **(**0.76**, **0.89**)	**< 0.001**
Falls in previous 12 months**	**1.44 **(**1.32**, **1.57**)	**< 0.001**	**1.23 **(**1.10**, **1.37**)	**< 0.001**
Fracture since 45 years	**3.49 **(**2.96**, **4.12**)	**< 0.001**	**2.63 **(**2.13**, **3.24**)	**< 0.001**
Family history of hip fracture	**1.34 **(**1.12**, **1.62**)	**0.002**	**1.40 **(**1.13**, **1.74**)	**0.002**
FRAX 10-year probability (MOF)*	**1.26 **(**1.16**, **1.36**)	**< 0.001**		
FRAX 10-year probability (hip fracture)*	**1.18 **(**1.09**, **1.27**)	**< 0.001**		
Number of comorbidities**	**1.20 **(**1.13**, **1.27**)	**< 0.001**	**1.08 **(**1.01**, **1.15**)	**0.033**

Ordinal logistic regression models were used with the 5-level variable for self-perceived fracture risk as the outcome

Significant associations (*p* < 0.05) are highlighted in bold. All characteristics were ascertained at baseline

^*^Odds ratio per standard deviation increase

^**^Odds ratio per higher category of characteristic

In sensitivity analyses among participants with osteoporosis, currently taking AOM and with either of these conditions, many associations were not significant, perhaps due to the reduction in sample size. However, the following characteristics associated with SPR in the main analysis were also significant (*p* < 0.05) or had a trend towards significance (*p* ≤ 0.071) in sensitivity analyses (Supplementary Table S1): physical activity; currently taking calcium; and having a fracture since aged 45 years.

### Cluster Analysis of Participant Characteristics

The four-cluster solution was optimal according to the BIC criterion; the number of participants in each cluster ranged from 459 to 904.

Descriptive statistics for the participant characteristics according to each cluster are shown in Table 4. Compared to the other clusters, Cluster 1 had a greater proportion of women with the following characteristics: current use of AOM (35.4% vs ≤ 4.5% in other clusters), calcium supplements (97.3% vs ≤ 1.1%) and Vitamin D supplements (51.5% vs ≤ 13.1%); and a fracture since age 45 years (33.7% vs ≤ 27.5%). Although not used in the cluster analysis algorithm, the proportion with higher SPR was also much higher in Cluster 1 (32.9%) compared to other clusters (≤ 11.2%).

**Table 4:** Participant characteristics according to each cluster

Participant characteristic	Cluster1 (*n* = 489)	Cluster2 (*n* = 904)	Cluster3 (*n* = 730)	Cluster4 (*n* = 459)
Age (years)*	69.3 (8.6)	65.1 (4.9)	76.0 (7.0)	59.1 (2.9)
Self-reported height (cm)*	161.7 (6.8)	163.0 (6.3)	160.3 (6.5)	163.7 (6.0)
Self-reported weight (kg)*	64.8 (10.9)	70.0 (12.7)	68.4 (13.2)	69.0 (13.0)
BMI (kg/m^2^)*	24.8 (4.0)	26.4 (4.6)	26.6 (5.0)	25.7 (4.7)
Self-perceived fracture risk				
Lower	96 (19.6%)	284 (31.4%)	217 (29.7%)	149 (32.5%)
Similar	232 (47.4%)	538 (59.5%)	431 (59.0%)	276 (60.1%)
Higher	161 (32.9%)	82 (9.1%)	82 (11.2%)	34 (7.4%)
Current smoker	22 (4.5%)	59 (6.5%)	41 (5.6%)	27 (5.9%)
Alcohol consumption				
None	131 (26.8%)	212 (23.5%)	322 (44.1%)	84 (18.3%)
1–6	197 (40.3%)	410 (45.4%)	285 (39.0%)	195 (42.5%)
7–13	129 (26.4%)	205 (22.7%)	96 (13.2%)	131 (28.5%)
14–20	30 (6.1%)	66 (7.3%)	23 (3.2%)	41 (8.9%)
> 20	2 (0.4%)	11 (1.2%)	4 (0.5%)	8 (1.7%)
Physically active compared to others				
Not at all	19 (3.9%)	12 (1.3%)	31 (4.2%)	9 (2.0%)
A little	75 (15.3%)	113 (12.5%)	173 (23.7%)	68 (14.8%)
Somewhat	257 (52.6%)	495 (54.8%)	322 (44.1%)	233 (50.8%)
Very	138 (28.2%)	284 (31.4%)	204 (27.9%)	149 (32.5%)
Educational attainment				
Below GCSE	156 (31.9%)	222 (24.6%)	427 (58.5%)	84 (18.3%)
GCSE	158 (32.3%)	356 (39.4%)	164 (22.5%)	169 (36.8%)
A-level	80 (16.4%)	142 (15.7%)	81 (11.1%)	72 (15.7%)
Degree	95 (19.4%)	184 (20.4%)	58 (7.9%)	134 (29.2%)
Current use of AOM	173 (35.4%)	16 (1.8%)	33 (4.5%)	4 (0.9%)
Ever used oestrogen/HRT	185 (37.8%)	472 (52.2%)	111 (15.2%)	176 (38.3%)
Currently taking calcium	476 (97.3%)	0 (0.0%)	8 (1.1%)	0 (0.0%)
Currently taking Vit D	252 (51.5%)	118 (13.1%)	71 (9.7%)	57 (12.4%)
Years since menopause				
Less than 10 years	71 (14.5%)	0 (0.0%)	0 (0.0%)	459 (100.0%)
10–19 years	162 (33.1%)	685 (75.8%)	12 (1.6%)	0 (0.0%)
20–29 years	141 (28.8%)	217 (24.0%)	326 (44.7%)	0 (0.0%)
30 or more years	115 (23.5%)	2 (0.2%)	392 (53.7%)	0 (0.0%)
Falls in previous 12 months				
None	288 (58.9%)	608 (67.3%)	435 (59.6%)	321 (69.9%)
Once	125 (25.6%)	206 (22.8%)	185 (25.3%)	78 (17.0%)
2 times or more	76 (15.5%)	90 (10.0%)	110 (15.1%)	60 (13.1%)
Fracture since 45 years	165 (33.7%)	90 (10.0%)	201 (27.5%)	31 (6.8%)
Family history of hip fracture	84 (17.2%)	130 (14.4%)	83 (11.4%)	74 (16.1%)
Number of comorbidities				
0	124 (25.4%)	271 (30.0%)	100 (13.7%)	167 (36.4%)
1	144 (29.4%)	319 (35.3%)	171 (23.4%)	152 (33.1%)
2	110 (22.5%)	207 (22.9%)	229 (31.4%)	93 (20.3%)
3	72 (14.7%)	88 (9.7%)	127 (17.4%)	32 (7.0%)
4+	39 (8.0%)	19 (2.1%)	103 (14.1%)	15 (3.3%)

^*^Mean (SD)

The cluster analysis was restricted to participants with complete data for all variables that were used in the cluster analysis algorithm (*n* = 2582)

BMI was derived from self-reported height and weight

The silhouette coefficient of 0.1 indicated that the clustering was not substantial. However, the results show that higher SPR and the risk factors for this variable tend to cluster together.

### Associations Between SPR and DXA aBMD Parameters

The relationships between SPR and DXA aBMD parameters are presented in Table 5. Higher SPR was associated (*p* < 0.02) with subsequent lower aBMD of the total hip, femoral neck and total lumbar spine in unadjusted analysis; the association regarding femoral neck aBMD was robust to adjustment (*p* = 0.003), whereas for total hip it was reduced by around 30%. The total hip encompasses the whole of the proximal femur region. In these women, it may be that the adjustment for body size and weight would have a much greater effect on this region of interest than on the femoral neck, which is a defined ROI-size not determined by the size of the bone. Also, whilst the total hip was reduced by 30%, the difference remains, albeit of borderline significance using the arbitrary *p* < 0.05 as the cut-off (*p* = 0.058).

**Table 5:** Standard deviation difference in mean DXA aBMD parameters (95%CI) per higher band of self-perceived fracture risk at baseline

Parameter	Unadjusted		Adjusted*	
	Estimate (95% CI)	*p*	Estimate (95%CI)	*p*
Whole body total	− 0.09 (− 0.21,0.03)	0.143	− 0.09 (− 0.22,0.04)	0.172
Total hip	− **0.16 **(− **0.26**, − **0.05**)	**0.005**	− 0.11 (− 0.22,0.00)	0.058
Femoral neck	− **0.18 **(− **0.29**, − **0.08**)	**0.001**	− **0.18 **(− **0.29**, − **0.06**)	**0.003**
Total lumbar spine	− **0.13 **(− **0.24**, − **0.02**)	**0.018**	− 0.12 (− 0.24,0.00)	0.053

Self-perceived fracture risk was used as an ordinal variable with the following bands: ‘much lower’; ‘a little lower’; ‘about the same’; ‘a little higher’; and ‘much higher’

Significant associations (*p* < 0.05) are highlighted in bold

*DXA* dual-energy X-ray absorptiometry, *p p *value, *aBMD* areal bone mineral density

^*^Adjusted for age at time of DXA scan, follow-up time, height, weight-for-height residual, physical activity, smoking status, alcohol consumption, education, time since last menstrual cycle, use of anti-osteoporosis medication, calcium and vitamin D supplements, and oestrogen/hormone replacement therapy (pill/skin patch)

### Associations Between SPR and Radial HRpQCT Parameters

The associations between SPR and radial HRpQCT parameters are presented in Table 6. Higher SPR was associated with lower trabecular volumetric density and number, and higher trabecular separation in unadjusted and adjusted analysis (*p* < 0.03).

**Table 6:** Standard deviation difference in mean HRpQCT parameters (95% CI) per higher band of self-perceived fracture risk at baseline

HRpQCT parameter	Unadjusted		Adjusted*	
	Estimate (95% CI)	*p*	Estimate (95% CI)	*p*
Radius				
Total area	− 0.03 (− 0.16,0.09)	0.609	0.02 (− 0.11,0.15)	0.783
Trabecular area	− 0.01 (− 0.13,0.12)	0.933	0.04 (− 0.09,0.17)	0.526
Cortical area	− 0.12 (− 0.24,0.01)	0.068	− **0.14 **(− **0.27**,**0.00**)	**0.047**
Cortical thickness	− 0.09 (− 0.21,0.04)	0.159	− 0.12 (− 0.26,0.02)	0.090
Cortical volumetric density	− 0.02 (− 0.14,0.11)	0.767	− 0.09 (− 0.23,0.04)	0.172
Cortical porosity	− 0.09 (− 0.21,0.04)	0.176	0.00 (− 0.14,0.14)	0.997
Cortical pores diameter	− 0.03 (− 0.15,0.10)	0.682	− 0.01 (− 0.16,0.14)	0.906
Trabecular volumetric density	− **0.16 **(− **0.28**, − **0.04**)	**0.010**	− **0.16 **(− **0.31**, − **0.02**)	**0.027**
Trabecular number	− **0.18 **(− **0.31**, − **0.06**)	**0.004**	− **0.19 **(− **0.33**, − **0.04**)	**0.010**
Trabecular thickness	− 0.04 (− 0.17,0.08)	0.499	− 0.05 (− 0.20,0.10)	0.522
Trabecular separation	**0.18 **(**0.06**,**0.30**)	**0.004**	**0.18 **(**0.04**,**0.33**)	**0.011**
Tibia				
Total area	− 0.01 (− 0.12,0.10)	0.852	0.00 (− 0.10,0.11)	0.953
Trabecular area	0.02 (− 0.09,0.13)	0.745	0.03 (− 0.08,0.13)	0.636
Cortical area	− **0.15 **(− **0.25**, − **0.04**)	**0.008**	− **0.12 **(− **0.23**, − **0.01**)	**0.038**
Cortical thickness	− **0.13 **(− **0.24**, − **0.03**)	**0.015**	− 0.10 (− 0.21,0.02)	0.093
Cortical volumetric density	− 0.06 (− 0.17,0.05)	0.287	− 0.07 (− 0.18,0.05)	0.240
Cortical porosity	0.00 (− 0.11,0.11)	0.952	0.03 (− 0.10,0.15)	0.682
Cortical pores diameter	− 0.01 (− 0.12,0.10)	0.832	0.02 (− 0.11,0.14)	0.791
Trabecular volumetric density	− **0.16 **(− **0.27**, − **0.06**)	**0.003**	− **0.14 **(− **0.26**, − **0.01**)	**0.036**
Trabecular number	− 0.09 (− 0.20,0.02)	0.109	− **0.13 **(− **0.26**, − **0.01**)	**0.035**
Trabecular thickness	− **0.11 **(− **0.22**, − **0.01**)	**0.040**	− 0.03 (− 0.15,0.10)	0.688
Trabecular separation	0.11 (0.00,0.22)	0.055	**0.14 **(**0.02**,**0.26)**	**0.027**

Self-perceived fracture risk was used as an ordinal variable with the following bands: ‘much lower’; ‘a little lower’; ‘about the same’; ‘a little higher’; and ‘much higher’

Significant associations (*p* < 0.05) are highlighted in bold

*pp *value; *HRpQCT* high resolution peripheral quantitative computed tomography

^*^Adjusted for age at time of HRpQCT scan, follow-up time, height, weight-for-height residual, physical activity, smoking status, alcohol consumption, education, time since last menstrual cycle, use of anti-osteoporosis medication, calcium and vitamin D supplements, and oestrogen/hormone replacement therapy (pill/skin patch)

### Associations Between SPR and Tibial HRpQCT Parameters

The associations between SPR and tibial HRpQCT parameters are also presented in Table 6. Higher SPR was associated with lower cortical area and thickness as well as lower trabecular volumetric density and thickness in unadjusted analysis (*p* < 0.05); relationships for cortical area and trabecular volumetric density were robust in adjusted analysis (*p* < 0.04). Higher SPR was related to higher trabecular separation in adjusted analysis (*p* = 0.027) and associations before adjustment were borderline significant (*p* = 0.055). When additionally adjusted for total hip aBMD, no associations regarding radial or tibial HRpQCT parameters were robust.

### Sensitivity Analysis

In this subgroup, 69 women had a fracture since age 45 years, 31 were using AOM, 63 had a family history of hip fracture, and 141 women had at least one of these characteristics. These groups have been identified as higher risk and this prior knowledge/experience is likely to increase their SPR score, and may have led to previous BMD testing. We were therefore interested to investigate the associations between higher SPR and aBMD and HRpQCT parameters in groups where participants with prior fracture, AOM use, family history of hip fracture and any of these three characteristics were excluded (data not shown). When each of these four sets of exclusions were applied, higher SPR was associated with lower femoral neck aBMD in unadjusted and adjusted analysis. When women on AOM at baseline were excluded, higher SPR remained associated with lower radial trabecular number and higher trabecular separation both before and after adjustments. When women with previous fractures were excluded, higher SPR remained associated with lower radial trabecular number and higher trabecular separation in adjusted analyses; relationships were borderline significant when those with a family history of hip fracture were excluded. Higher SPR was related to lower tibial trabecular volumetric density when women with family history of hip fracture were excluded; in the other sets of sensitivity analyses, no other associations regarding tibial parameters were robust in both unadjusted and adjusted analysis. When all three exclusions were applied, no tibial or radial associations were robust.

## Discussion

In this study, we have identified personal characteristics associated with self-perception of risk of fracture. A cluster analysis of baseline participant characteristics identified one cluster, in which higher SPR, prior history of fracture since age of 45, current use of AOM, vitamin D and calcium supplementation clustered together. Hence this seems to identify women who, through prior fracture experience, have initiated and remained on therapy and acknowledge their higher fracture risk. However, despite greater use of anti-osteoporosis medications, a higher SPR was still related to impaired bone density and microarchitecture measured a median of 7.5 years later. Associations were similar even when separately excluding the following groups of participants: previously experienced a fracture since age 45; reported a family history of hip fracture; and taking AOM. Although associations regarding tibial and radial HRpQCT parameters were attenuated when participants with any of these three characteristics were excluded, this could have been due to the reduction in sample size and robust associations between higher SPR and lower femoral neck aBMD remained after these exclusions.

To our knowledge this is the first time that associations between SPR and DXA aBMD and HRpQCT parameters among postmenopausal women have been examined, and suggests that women can correctly identify personal factors associated with heightened osteoporosis risk, but despite uptake of AOM, that risk remains elevated at around 7.5 years later. Findings from this study demonstrated that higher SPR bands are related to a decrease in areal BMD at the femoral neck and lower tibial trabecular volumetric density. There is evidence to suggest that a 1SD decrease in BMD is associated with a 1.5-threefold times higher fracture risk [13]. Our data suggests that it is likely that there will be increased fracture risk in women with higher SPR as they continue to lose bone and age.

There are limitations to our study. These are observational data that demonstrate associations, but not causality, and need to be tested in other populations. Secondly, the SPR questionnaire has not been validated. Finally, there is no information available if the participants had a DXA scan performed prior. Women who have had a prior fracture or took bone-specific treatment may well have had a DXA scan. It would not be unexpected that those participants rated their SPR as higher compared to other women of the same age. Those women were likely to integrate the bone protective behaviour and measures into their daily life resulting in a ‘self-fulfilling prophesy’. However, even if it is taken into account, we still observed lower aBMD and less favourable HRpQCT parameters around 7 years later in this group. In many ways, this group represent the ‘best case’ scenario of osteoporosis care in that women have been identified as osteoporotic, recognise this diagnosis and remain on therapy to counteract this risk. The situation in many clinical cases may be much worse. Longer follow up of this group could be highly beneficial.

In the current study, the higher SPR was associated with higher FRAX scores for MOF and hip fracture. However, SPR of osteoporosis and osteoporotic fractures has been reported to be underestimated in postmenopausal women worldwide. Rothmann et al. observed that women participating in the Risk-Stratified Osteoporosis Strategy Evaluation (ROSE) study underestimated their fracture risk compared to the risk estimated by FRAX [14]. Similarly, findings from GLOW showed that women at increased fracture risk generally perceive their risk to be lower or about the same as women of the same age [8, 15]. Furthermore, it was previously demonstrated in GLOW that SPR of fracture does capture some aspect of fracture risk not currently measured using the conventional fracture prediction tool FRAX [5]. The perception of personal risk has been shown to modify an individual’s behaviour related to their bone health [5, 7]. Heightened self-perceived risks of osteoporosis and fracture significantly increases the likelihood of seeking medical advice hence, increasing the chances, in appropriate individuals, of being given a diagnosis of osteoporosis—a well known predictor of treatment initiation [7]. Moreover, heightened self-perceived risks of fracture are known to be associated with BMD testing.

Although the positive effect of risk perception on BMD testing has been previously described, the analysis of the relationship between the results of bone microarchitecture parameters and fracture risk perception is novel. There is evidence that other factors independent of aBMD, including skeletal properties of trabecular microstructure examined by HRpQCT, contribute to fracture risk [16–18]. This study suggests that there is an association between SPR and bone microarchitecture. Taking osteoporosis medications was strongly associated with a higher self-perceived fracture risk in this study. This concurs with findings from a cross-sectional analysis of GLOW where women with higher SPR were more likely to report AOM use than women with lower SPR [5]. Barcenilla-Wong et al. also reported that elevated self-perceived risk of fracture increases the likelihood of taking AOM prospectively [7].

In conclusion, we have identified individual characteristics correlated with higher SPR, considered how they cluster together and studied relationships between SPR and subsequent objectively assessed bone health. This is particularly notable as previous research has suggested that while women often underestimate fracture risk, a higher SPR is associated with health seeking behaviour and better compliance with OP medication, as we observed in this study. An exploration of SPR through further studies, including qualitative work, may allow development of novel fracture prediction methods, and strategies to reduce fracture risk.

## Electronic supplementary material

Below is the link to the electronic supplementary material.

Supplementary file1 (DOCX 14 kb)
