# Supplementary material for: Self-perceived Fracture Risk in the Global Longitudinal Study of Osteoporosis in Women: Its Correlates and Relationship with Bone Microarchitecture
Source: Calcif Tissue Int. 2020 Mar 5;106(6):625–36. doi: 10.1007/s00223-020-00680-9 (PMC7188698; doi:10.1007/s00223-020-00680-9)
Supplement: Supplementary file 1 — Supplementary file1 (DOCX 14 kb) [file 223_2020_680_MOESM1_ESM.docx]

| **Supplementary Table 1: Odds ratios (OR) for having a higher category of self-perceived fracture risk for the presence versus absence of each characteristic among participants with an osteoporosis diagnoses and currently using anti-osteoporotic medications (AOM)** | | | | | | |
| --- | --- | --- | --- | --- | --- | --- |
| **Characteristic** | **Osteoporosis diagnosis** | | **Using AOM** | | **Osteoporosis or using AOM** | |
|  | **OR (95% CI)** | **P-value** | **OR (95% CI)** | **P-value** | **OR (95% CI)** | **P-value** |
| Age* | 0.91 (0.75,1.11) | 0.350 | 0.78 (0.63,0.97) | 0.022 | 0.88 (0.74,1.04) | 0.136 |
| Self-reported height* | 0.98 (0.81,1.19) | 0.875 | 1.01 (0.82,1.23) | 0.952 | 0.96 (0.81,1.13) | 0.588 |
| Weight-for-height residual* | 0.89 (0.74,1.06) | 0.189 | 0.86 (0.70,1.05) | 0.140 | 0.90 (0.77,1.06) | 0.220 |
| Current smoker | 0.95 (0.48,1.90) | 0.887 | 1.89 (0.77,4.64) | 0.165 | 1.01 (0.53,1.90) | 0.981 |
| Alcohol consumption** | 1.07 (0.87,1.31) | 0.545 | 0.93 (0.75,1.14) | 0.474 | 0.95 (0.80,1.13) | 0.579 |
| Physically active compared to others of similar age** | 0.70 (0.56,0.86) | 0.001 | 0.67 (0.53,0.84) | 0.001 | 0.67 (0.56,0.81) | <0.001 |
| Educational attainment** | 1.06 (0.90,1.25) | 0.465 | 1.09 (0.92,1.30) | 0.323 | 1.09 (0.94,1.26) | 0.249 |
| Current use of anti-osteoporotic medication | 2.60 (1.79,3.79) | <0.001 | N/A | N/A | N/A | N/A |
| Ever used estrogen/hormone replacement therapy | 1.41 (0.96,2.09) | 0.083 | 1.49 (0.98,2.27) | 0.065 | 1.35 (0.96,1.89) | 0.086 |
| Currently taking calcium supplements | 1.48 (1.02,2.13) | 0.038 | 1.49 (0.98,2.27) | 0.062 | 1.42 (1.03,1.95) | 0.033 |
| Currently taking Vit D/multivitamin with Vit D | 1.57 (1.04,2.36) | 0.032 | 1.49 (0.95,2.32) | 0.080 | 1.48 (1.03,2.13) | 0.033 |
| Years since menopause** | 0.96 (0.79,1.17) | 0.682 | 0.99 (0.80,1.22) | 0.908 | 0.99 (0.83,1.17) | 0.895 |
| Falls in previous 12 months** | 1.10 (0.89,1.36) | 0.370 | 1.10 (0.86,1.39) | 0.455 | 1.19 (0.99,1.44) | 0.066 |
| Fracture since 45 years | 1.85 (1.28,2.66) | 0.001 | 1.43 (0.97,2.12) | 0.071 | 1.79 (1.30,2.47) | <0.001 |
| Family history of hip fracture | 1.09 (0.68,1.77) | 0.717 | 1.03 (0.61,1.73) | 0.922 | 1.18 (0.77,1.82) | 0.453 |
| FRAX 10-year probability (MOF)* | 1.16 (0.91,1.47) | 0.227 | 0.98 (0.77,1.25) | 0.879 | 1.08 (0.88,1.32) | 0.466 |
| FRAX 10-year probability (hip fracture)* | 1.11 (0.87,1.41) | 0.420 | 0.92 (0.72,1.18) | 0.518 | 1.02 (0.83,1.26) | 0.858 |
| Number of comorbidities** | 0.89 (0.76,1.04) | 0.129 | 1.00 (0.84,1.19) | 0.965 | 0.94 (0.82,1.08) | 0.396 |
| Ordinal logistic regression models were used with the 5-level variable for self-perceived fracture risk as the outcome. All characteristics were ascertained at baseline  *Odds ratio per standard deviation increase **Odds ratio per higher category of characteristic | | | | | | |
